# Enhancement of trapping efficiency by utilizing a hollow sinh-Gaussian beam

**DOI:** 10.1038/s41598-019-46716-5

**Published:** 2019-07-15

**Authors:** Zhirong Liu, Xun Wang, Kelin Hang

**Affiliations:** Department of Applied Physics, East China Jiaotong University, Nanchang, 330013 China

**Keywords:** Micro-optics, Optical manipulation and tweezers

## Abstract

Propagation properties and optical forces upon a Rayleigh dielectric sphere for a newly proposed hollow sinh-Gaussian beam (HsGB) are intensively investigated. In view of the targeted laser beam’s unique tight focusing properties that a significantly sharp, peak-centered, and adjustable intensity distribution would be produced in the focal vicinity, the tightly focused HsGB could be exploited to trap and manipulate nano-sized dielectric spheres with high-refractive index in the focal region. The interesting and meaningful features for the novel HsGB mainly include that, compared with the conventional fundamental Gaussian beams under the same optical power, the tightly focused HsGB has much higher intensity gradient and deeper potential well through optimizing targeted laser beam’s parameters. Theretofore, the novel HsGB optical tweezers could drastically enhance its trapping efficiency. Finally, the trapping stability conditions are discussed in detail. The analytical and numerical results obtained here could provide a directive suggestion for researchers in optimizing experimental parameters in constructing a novel HsGB tweezers and making use of a HsGB.

## Introduction

Recently, dark-hollow beams (DHBs) have attracted considerable research interest and extensive attention due to its interesting characteristics and potential usefulness in application fields mainly including atomic optics, optical communication, and optical manipulation^1–3^. Till now, several methods have been applied to generate DHBs^4–8^, and meanwhile, several theoretical models have been proposed to describe DHBs^9–11^. In 2012, Sun *et al*. introduced a novel mathematical model called hollow sinh-Gaussian beams (HsGBs) to depict DHBs, and the targeted laser beams’ propagation characteristics in free space were also examined^12^. Moreover, owing to the HsGBs’ special properties, especially for its central hollow region, they further predicted that HsGBs might find potential applications in atom guiding and trapping^12^. In recent years, several works on tight focusing and application of various polarized HsGBs’ were reported^13–16^. For instance, Lin *et al*. considered the radially polarized HsGBs’ tight focusing performance and showed that high beam quality and subwavelength focusing could be achieved, which was expected be applied to achieve focusing with superresolution^13^; Liu *et al*. examined the tightly focused azimuthally polarized HsGBs and found that a steeper depleting pattern and an exciting pattern without side lobes could be produced, which was helpful to achieve super resolution in STED microscopy^14^; Senthilkumar *et al*. numerically investigated the tight focusing properties of spirally polarized HsGBs and revealed that many novel focal patterns including flattop profile, focal hole axially separated focal spots and focal spot with long focal depth could be evolved, which tunable focal patterns were expected to be useful for optical manipulation of micro particles^15^; Zou *et al*. studied the propagation properties of a single HsGB and their interactions through the quadratic-index medium^16^.

As we know that photon carries both linear and angular momentums. Through momentum exchange light field would exert radiation (optical) force and torque on objects it encounters. Since the first demonstration of acceleration and trapping of particles by radiation pressure^17^, especially a single-beam gradient force optical trap (i.e., optical tweezers) for dielectric particles was first observed in experiment^18^, researches on optical tweezers have made great progress. Through the unremitting efforts of scientific researchers, nowadays, optical tweezers have become a powerful and indispensable tool in the following areas such as physics, chemistry, and biology^19^. Researchers have revealed that optical forces are closely related to the structure of the focused laser fields. And till now, a great variety of structured light fields have been generated and tailored to manipulate various tiny objects^19–28^.

In this work, we first derived the propagation formula for the HsGBs through an ABCD optical system, and then analyzed the HsGBs’ tight focusing properties. Subsequently, we exploited the highly focused HsGBs to trap and manipulate nano-sized dielectric spheres with high-refractive index in the focal region. The interesting and meaningful features for the novel HsGBs mainly included that, compared with the conventional fundamental Gaussian beams under the same optical power, the tightly focused HsGBs have much higher intensity gradient and deeper potential well through optimizing targeted laser beam’s parameters. Theretofore, the novel HsGB optical tweezers can greatly enhance its trapping efficiency. Finally, we analyzed the trapping stability conditions.

## Propagation of HsGBs Through an ABCD Optical System

According to the novel mathematical model of dark-hollow beams proposed by Sun *et al*., the electric field of HsGBs in the original plane *z* = 0 takes the form^12^

1 $${E}_{n}(r,0)={G}_{0}{\sinh }^{n}(\frac{r}{{w}_{0}})\exp (-\frac{{r}^{2}}{{w}_{0}^{2}}).$$

In Eq. (1), *G*_0_ denotes a constant related to the laser beams power *P*_0_, and *n* (*n* = 0, 1, 2, ⋅⋅⋅) represents the order of HsGBs. Here, it is noted that the central dark size and the construction of HsGBs could conveniently be controlled by the laser beams order. Also, one can note that, for the case *n* = 0, Eq. (1) represents the fundamental Gaussian beams with waist size *w*_0_; for the case *n* ≥ 1, Eq. (1) stands for the so-called hollow sinh-Gaussian beams (HsGBs). It is noted from Eq. (1) that HsGBs could be considered as superposition of a series of eccentric Gaussian beam, of which characteristic would be determined by the order *n* and waist size *w*_0_.

**Figure 1:**
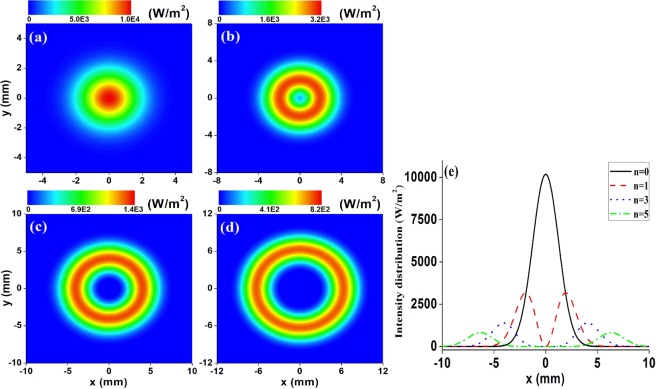
Contour graphs of the normalized intensity distribution of HsGBs with several orders n: (**a**) n = 0, (**b**) n = 1, (**c**) n = 3, (**d**) n = 5, and (**e**) the corresponding cross intensity lines at *y* = 0. The remaining parameters are *P*_0_ = 100 mW, *f* = 25 mm, and *w*_0_ = 10 mm.

In order to visualize the profiles of HsGBs, Fig. 1 illustrates the contour graphs of the normalized intensity distribution of HsGBs with several orders n. All the contour graphs and curves in Fig. 1 have been normalized by a fixed optical power *P*_0_ = 100 mW. It is clearly seen from Fig. 1 that, for the case n = 0, the irradiance profile of fundamental Gaussian beam presents a peak-centered configuration; for the case n ≥ 1, the irradiance profile of HsGB presents a single bright ring configuration, and furthermore, the central dark size increases as n increases. Therefore, by selecting appropriate laser beams order, one may obtain HsGBs with ideal intensity distribution.

For the convenience of integration, Eq. (1) was to be rewritten in the form

2 $${E}_{n}(r,0)={G}_{0}\sum _{m=0}^{n}{a}_{m}{b}_{m}\exp [\frac{-{(r+{c}_{m})}^{2}}{{w}_{0}^{2}}].$$

In Eq. (2), several coefficients are, respectively, given by

3 $${a}_{m}={(-1)}^{m}{2}^{-n}(\begin{array}{c}n\\ m\end{array}),\,{b}_{m}=\exp [(m-\frac{n}{2}{)}^{2}],\,{c}_{m}={w}_{0}(m-\frac{n}{2}),$$

where $$(\begin{array}{c}n\\ m\end{array})$$ is a binomial coefficient, and −*c*_*m*_ denotes a center parameter. Within the framework of the paraxial approximation, propagation of laser beams through a paraxial ABCD optical system could be described by the generalized Huygens-Fresnel diffraction integral, known as Collins formula, which takes the following form in a cylindrical coordinate system^29^

4 $$\begin{array}{c}{E}_{n}(r,z)=\frac{i}{\lambda B}\exp (\,-\,ikz)\\ \,\,\,\,\times {\int }_{0}^{2\pi }{\int }_{0}^{\infty }{E}_{n}(r^{\prime} ,0)\exp \{-\frac{ik}{2B}[A{r}^{^{\prime} 2}-2rr^{\prime} \cos (\theta -\theta ^{\prime} )+D{r}^{2}]\}r^{\prime} dr^{\prime} d\theta ^{\prime} .\end{array}$$

In Eq. (4), *E*_*n*_(*r*′, 0) and *E*_*n*_(*r*, *z*) denote the electric fields in the input and output planes, respectively. *r*′, *θ*′ and *r*, *θ* are the radial and azimuthal angle coordinates in the input and output planes, respectively. *z* is the axial distance between the input and output planes along the optical axis. *k* is the wave number related to the wavelength *λ* by *k* = 2*π*/*λ*. A, B, C and D are the transfer matrix elements of the optical system. Substituting Eqs (2) and (3) into Eq. (4), and recalling the following integral formulae^30^:

5 $${J}_{0}(t)=\frac{1}{2\pi }{\int }_{0}^{2\pi }\exp (it\,\cos \,\phi )d\phi ,$$

6 $$\begin{array}{rcl}{\int }_{0}^{\infty }{t}^{\mu }\exp (\,-\,{a}^{2}{t}^{2}){J}_{v}(pt)dt & = & \Gamma (\frac{\mu +v+1}{2})\frac{{p}^{v}}{{2}^{v+1}{a}^{\mu +v+1}\Gamma (v+1)}\\  &  & \times \,{}_{1}F_{1}(\frac{\mu +v+1}{2},v+1;-\,\frac{{p}^{2}}{4{a}^{2}}),\end{array}$$

where *J*_*v*_(*x*) stands for the *v*-order Bessel function of the first kind, *Γ*(*x*) denotes the gamma function, and _1_*F*_1_(*a*, *b*; *x*) is the confluent hypergeometric function, after tedious but straightforward integration, one could get

7 $$\begin{array}{c}{E}_{n}(r,z)=\frac{i{G}_{0}k}{2B}\exp (\,-\,ikz)\exp (-\frac{ikD{r}^{2}}{2B})\\ \,\,\,\,\times \sum _{m=0}^{n}\sum _{s=0}^{\infty }{(-1)}^{m}{2}^{-n}(\begin{array}{c}n\\ m\end{array})\frac{{(n-2m)}^{s}}{{w}_{0}^{s}\cdot s!}\\ \,\,\,\,\times \Gamma (1+\frac{s}{2})\cdot {(\frac{1}{{w}_{0}^{2}}+\frac{ikA}{2B})}^{-1-s/2}\cdot {}_{1}F_{1}(1+\frac{s}{2},1;-\,\frac{{(\frac{kr}{2B})}^{2}}{(\frac{1}{{w}_{0}^{2}}+\frac{ikA}{2B})}).\end{array}$$

Equation (7) is the general propagation formula for HsGBs through an ABCD optical system, by which the novel laser beams propagation and transformation through various optical systems could be treated conveniently.

## Tight Focusing Properties of HsGBs

**Figure 2:**
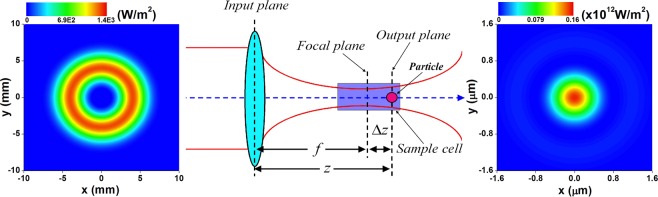
Schematic of the focusing optical system. Left and right plots are the intensity distribution of the 3rd-order HsGB in the input and output planes, respectively. The remaining parameters are *P*_0_ = 100 mW, *f* = 25 mm, and *w*_0_ = 10 mm.

In order to investigate HsGBs’ tight focusing properties, let us consider the laser beams propagate through a thin lens system as shown in Fig. 2. According to the principle of Matrix Optics^31^, the transfer matrix between the input and output planes could be given by

8 $$[\begin{array}{cc}A & B\\ C & D\end{array}]=[\begin{array}{cc}1 & f+\Delta z\\ 0 & 1\end{array}][\begin{array}{cc}1 & 0\\ -1/f & 1\end{array}]=[\begin{array}{cc}1-z/f & z\\ -1/f & 1\end{array}].$$

In Eq. (8) *f* is the focal length, Δ*z* is the distance between the geometrical focus and the output plane, and *z* = *f* + Δ*z* is the axial distance from the output (or reference) plane to the input plane. Substituting Eq. (8) into Eq. (7), one could obtain the normalized intensity distribution of HsGBs through a lens optical system. In Fig. 2, the left and right plots show the intensity distributions of the 3rd-order HsGBs in the input and output planes, respectively. It is clearly seen from Fig. 2 that through the focusing system the original millimeter-sized and hollow laser beam turns to a submicro-sized and peak-centered configuration.

**Figure 3:**
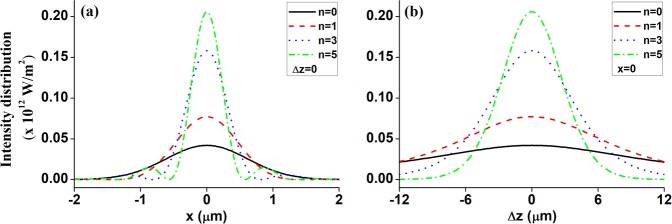
Intensity distribution for different ordered HsGBs near the focus in the transverse plane (**a**) Δ*z* = 0, and the longitudinal plane (**b**) *x* = 0, respectively. The remaining parameters are *P*_0_ = 100 mW, *f* = 25 mm and *w*_0_ = 10 mm.

To further examine the HsGB’s tight focusing properties in detail, Fig. 3 plots the intensity distribution of different ordered HsGBs near the focus both in the transverse plane (a) Δ*z* = 0, and the longitudinal plane (b) *x* = 0. The parameters are, respectively, selected as: *λ*_0_ = 0.514 μm, *w*_0_ = 10 mm, and *f* = 25 mm. For comparison convenience, the orders *n* of HsGBs are set as 0 (i.e., fundamental Gaussian beams), 1, 3 and 5, respectively. From Fig. 3, it is obvious that both the transverse (see Fig. 3(a)) and the longitudinal (see Fig. 3(b)) intensity distribution presents a significantly sharp and peak-centered configuration. Moreover, the focusing intensity distribution would be sensitively adjusted by the HsGBs’ order: increase the HsGBs’ order, and the peaks become dramatically sharper. Due to these focusing properties, the highly focused HsGBs might be exploited to trap and manipulate nano-sized dielectric particles with high-refractive index in the focal region.

## Optical Forces on a Rayleigh Dielectric Sphere Produced by Tightly Focused HsGBs

In this section, we investigate the optical forces exerted on a nano-sized dielectric sphere produced by the tightly focused HsGBs. For simplicity, the radius of sphere is assumed to be much smaller than the laser beam wavelength (*a* ≤ *λ*/20). In this situation, the Rayleigh approximation is valid to deal with the scattering between the focused laser field and the sphere, and the optical forces exerted on the objects mainly include gradient force ${\mathop{F}\limits^{\rightharpoonup }}_{grad}$ and scattering force ${\mathop{F}\limits^{\rightharpoonup }}_{scat}$. According to the refs^20,32^, the gradient force and the scattering force are, respectively, defined by

9 $${\mathop{F}\limits^{\rightharpoonup }}_{grad}(r,z)=\frac{2\pi {n}_{2}{a}^{3}}{c}(\frac{{n}_{1,2}^{2}-1}{{n}_{1,2}^{2}+2})\nabla I(r,z),$$

10 $${\mathop{F}\limits^{\rightharpoonup }}_{scat}(r,z)=\hat{z}\frac{{n}_{2}}{c}{C}_{pr}I(r,z),$$

with

11 $$I(r,z)=\frac{{n}_{2}{\varepsilon }_{0}c}{2}{|E(r,z)|}^{2},$$

12 $${C}_{pr}={C}_{scat}=\frac{8}{3}\pi {(ka)}^{4}{a}^{2}{(\frac{{n}_{1,2}^{2}-1}{{n}_{1,2}^{2}+2})}^{2},$$

where *a* is the sphere’s radius, *n*_1,2_ = *n*_1_/*n*_2_ stands for the relative index with *n*_1_ and *n*_2_, respectively, representing the refractive index of the sphere and the ambient, and c is the light field’s propagation speed in vacuum. It is pointed out that *C*_*pr*_ denotes the cross section for the optical pressure of the sphere, which is assumed to be equal to the scattering cross section *C*_*scat*_ for a dielectric sphere in the Rayleigh regime. Using Eqs (7–12), one could calculate the optical forces acting upon a Rayleigh dielectric sphere produced by highly focused HsGBs. Without loss of generality, in the following calculations we select the radius of sphere *a* = *λ*/20 = 25 nm, the high-refractive index of the nano-sized particle *n*_1_ = 1.59 (i.e., glass), and the index of surrounding medium *n*_2_ = 1.33 (i.e., water).

**Figure 4:**
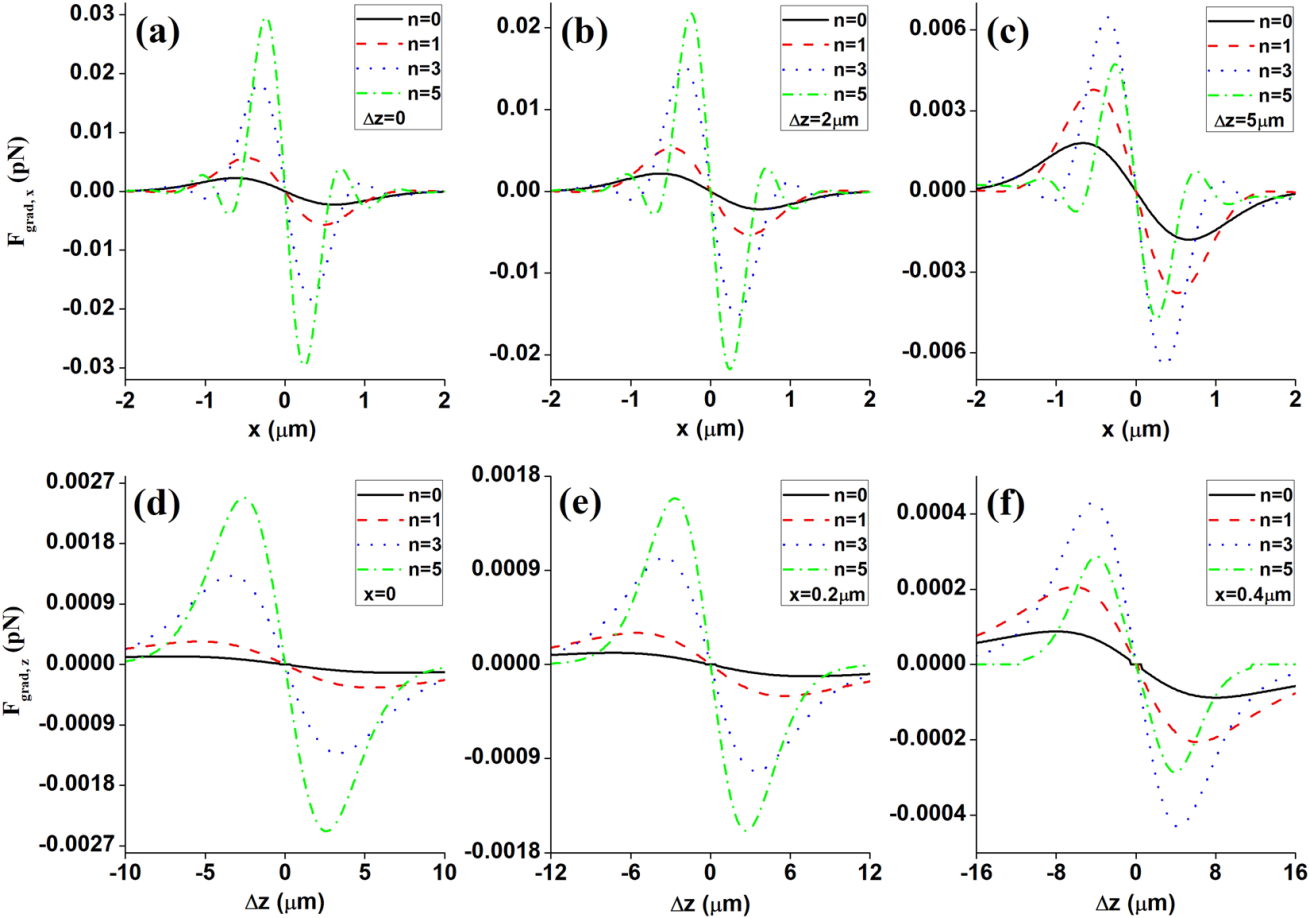
The transverse gradient force *F*_*grad*,*x*_ (**a**–**c**) and longitudinal gradient force *F*_*grad*,*z*_ (**d**–**f**) exerted upon a Rayleigh dielectric sphere produced by focused different ordered HsGBs near the focus at several planes: (**a**) Δ*z* = 0, (**b**) Δ*z* = 0.3 μm, (**c**) Δ*z* = 0.6 μm, (**d**) *x* = 0, (**e**) *x* = 0.1 μm, (**f**) *x* = 0.2 μm. The remaining parameters are *P*_0_ = 100 mW, *f* = 25 mm, *w*_0_ = 10 mm, *n*_2_ = 1.33, *n*_1_ = 1.59, and *a* = 25 nm.

Figure 4(a–c) illustrates the transverse gradient force ${\mathop{F}\limits^{\rightharpoonup }}_{grad,x}$, and Fig. 4(d–f) the longitudinal gradient force ${\mathop{F}\limits^{\rightharpoonup }}_{grad,z}$ exerted upon a Rayleigh dielectric sphere for the focused different ordered HsGBs in the focal vicinity. It is clearly found from Fig. 4 that one stable equilibrium point is distributed at the focus both in the transverse and longitudinal directions for high-refractive-index (*n*_1,2_ > 1) spheres (see Fig. 4(a,d)). It indicates that the focused HsGBs could be exploited to trap and manipulate spheres with refractive index larger than that of the surrounding medium. Moreover, from Fig. 4(a,d), one could also find that both the transverse and longitudinal gradient forces become much greater and sharper (i.e., the optical trap stiffness, which is defined by differentiating the gradient forces, is enhanced) when increase the HsGBs’ order *n*, which indicates that high-ordered HsGBs could greatly improve the optical tweezers’ optical trapping efficiency. Meanwhile, the optical trapping range would decrease when the HsGBs’ order *n* increases.

**Figure 5:**
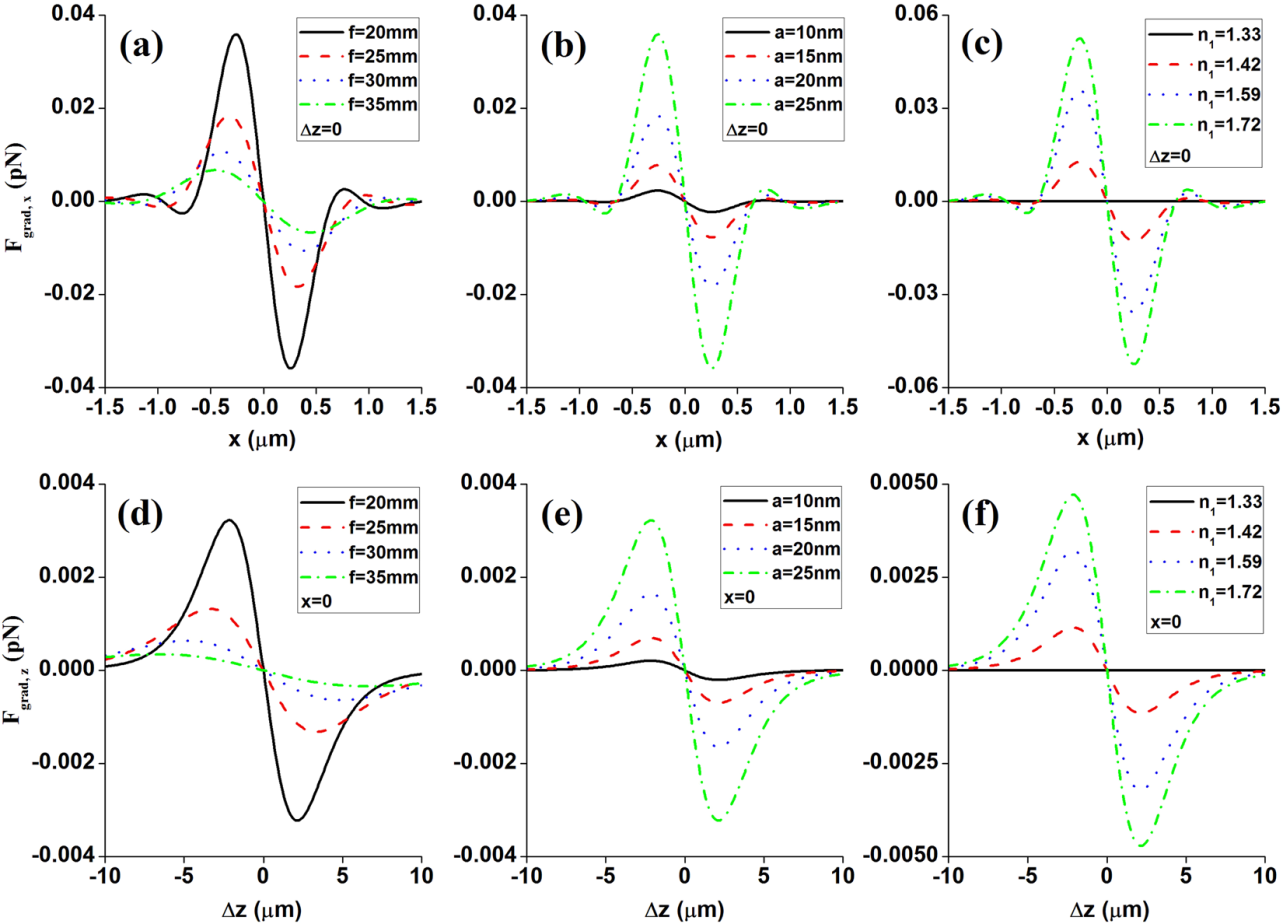
The changes of the transverse gradient forces (**a**–**c**) and longitudinal gradient forces (**d**–**f**) for several values of (**a**,**d**) the focal length *f*; (**b**,**e**) particle’s size *a*; (**c**,**f**) particle’s refractive index *n*_1_. The remaining parameter is *n* = 3.

In addition, it should be noted that the HsGB’s optical forces would be influenced by several factors, such as the laser beam power *P*_0_, laser beam size *w*_0_, focal length *f*, particle’s size *a*, and the relative refractive index *n*_1,2_ = *n*_1_/*n*_2_, etc. It is easily obtained from Eqs (9) and (10) that, the optical forces would increase when increase the laser beam power or reduce the laser beam size. To investigate the influence of the rest factors on the HsGB’s optical forces, Fig. 5 presents the changes of the transverse gradient forces (a–c) and the longitudinal gradient forces (d–f) for several values of focal length *f*, particle’s size *a*, and particle’s refractive index *n*_1_. It is clearly seen from Fig. 5(a,d) that the shorter the focal length is, the larger the optical forces would become. And it could also be found that when increase the particle’s size (see Fig. 5(b,e)), or increases the gap between the refractive index of the particle and that of the surrounding medium (see Fig. 5(c,f)), the HsGB’s optical forces would increase, and meanwhile, the optical trap stiffness would be significantly improved. In other words, shorter focal length, bigger particle size, and greater gap between the refractive index *n*_1_ of the particle and the refractive index *n*_2_ of the surrounding medium are all favorable factors for the optical trapping.

## Trapping Stability Analysis

Through the above analysis, one may have an image that tightly focused HsGBs could be exploited to trap and manipulate high-refractive-index Rayleigh dielectric spheres in the focal regime. In order to capture or manipulate a Rayleigh particle stably, there are still two necessary factors that the optical manipulation system has to fulfill^18^

13 $$R=|{\overrightarrow{F}}_{grad,z}|/|{\mathop{F}\limits^{\rightharpoonup }}_{scat}|\ge 1,$$

14 $${R}_{thermal}=\exp (\,-\,{U}_{{\rm{\max }}}/{k}_{B}T)\ll 1,$$

**Figure 6:**
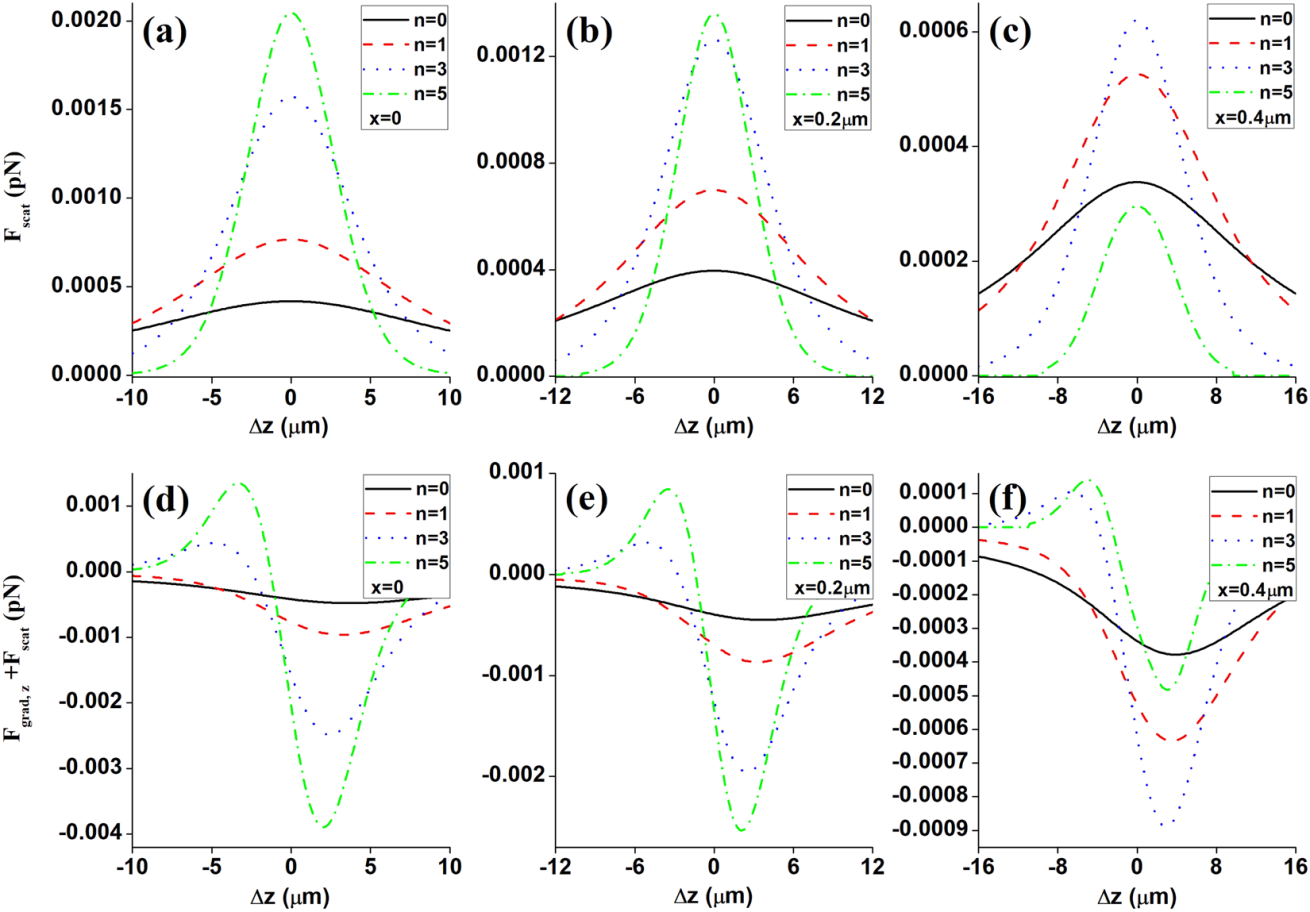
The scattering force ${\mathop{F}\limits^{\rightharpoonup }}_{scat}$ (**a**–**c**) and total longitudinal optical forces ${\mathop{F}\limits^{\rightharpoonup }}_{grad,z}$ + ${\mathop{F}\limits^{\rightharpoonup }}_{scat}$ exerted upon a Rayleigh dielectric sphere for the focused different ordered HsGBs near the focus at several planes: (**a**,**d**) *x* = 0, (**b**,**e**) *x* = 0.1 μm, and (**c**,**f**) *x* = 0.2 μm. The remaining parameters are *P*_0_ = 100 mW, *f* = 25 mm, *w*_0_ = 10 mm, *n*_2_ = 1.33, *n*_1_ = 1.59 and *a* = 25 nm.

Equation (13) stands for the first necessary factor, which indicates that the maximum axial gradient force should be greater than the maximum scattering force, where R is defined as the stability criterion. Figure 6(a–c) shows the scattering force exerted upon a Rayleigh dielectric sphere for the focused different ordered HsGBs near the focus at several planes. Comparing the longitudinal gradient force ${\mathop{F}\limits^{\rightharpoonup }}_{grad,z}$ (see Fig. 5(d–f)) and the scattering force ${\mathop{F}\limits^{\rightharpoonup }}_{scat}$ (see Fig. 6(a–c)) at the same position, the magnitude of ${\mathop{F}\limits^{\rightharpoonup }}_{grad,z}$ is much greater (about 19-times greater) than that of ${\mathop{F}\limits^{\rightharpoonup }}_{scat}$. Since ${\mathop{F}\limits^{\rightharpoonup }}_{grad,z}$ is dominated in the *z* direction, the total longitudinal optical forces (i.e., ${\mathop{F}\limits^{\rightharpoonup }}_{grad,z}$ + ${\mathop{F}\limits^{\rightharpoonup }}_{scat}$) would presents the restoring force characteristics, which could be verified by Fig. 6(d–f). Hence, the first stability criterion, i.e., Eq. (13) is fulfilled well. Secondly, to stably trap or manipulate a particle, the potential well, which is generated by the gradient force and defined by ${U}_{{\rm{\max }}}=\pi {\varepsilon }_{0}{n}_{2}^{2}{a}^{3}|({n}_{1,2}^{2}-1)/({n}_{1,2}^{2}+2)|\cdot |{E}_{{\rm{\max }}}{|}^{2}$, should surpass the particle’s kinetic energy. So, the second stability criterion could be expressed by Eq. (14), where *k*_*B*_ denotes the Boltzmann factor and *T* is the absolute temperature of the ambient. In our numerical examples, at room temperature of 300 K, for the high-refractive index particle (*n*_1_ = 1.59), the value of *R*_*thermal*_ at the maximum intensity position (*x* = 0, *y* = 0, Δ*z* = 0) is about *R*_*thermal*_ ≈ 0.055. Theretofore, the second stability criterion, i.e., Eq. (14) is also fulfilled very well. In all, through the above analyzing we could draw a conclusion that in order to obtain better trapping efficiency by HsGBs tweezers, one could select higher-order HsGBs and higher relative refractive index dielectric spheres with radius *a* < 25 nm.

## Conclusions

Based on the generalized Huygens-Fresnel diffraction integral, we first derived the analytical formula for a newly proposed hollow sinh-Gaussian beam (HsGB) propagating through an ABCD optical system, and then analyzed its tight focusing. Due to the targeted laser beam’s unique tight focusing properties that a significantly sharp, peak-centered, and adjustable intensity distribution would be produced in the focal vicinity, subsequently, then we exploited the highly focused HsGBs to trap and manipulate nano-sized dielectric spheres with high-refractive index in the focal region. The interesting and meaningful features for the novel HsGBs mainly include that, compared with the conventional fundamental Gaussian beams under the same optical power, the tightly focused HsGBs have much higher intensity gradient and deeper potential well through optimizing targeted laser beam’s parameters. Theretofore, the novel HsGB optical tweezers can greatly enhance its trapping efficiency. Finally, we analyzed the trapping stability conditions. The analytical and numerical results obtained here could provide a directive suggestion for researchers in optimizing experimental parameters in constructing a novel HsGB tweezers and making use of a HsGB.

## References

[1] Kuga T (1997). Novel optical trap of atoms with a doughnut beam. Phys. Rev. Lett..

[2] Ito H (1996). Laser spectroscopy of atoms guided by evanescent waves in micron-sized hollow optical fibers. Phys. Rev. Lett..

[3] Vetelino FES, Andrews LC (2004). Annular Gaussian beams in turbulent media. Proc. SPIE.

[4] Wang X, Littman MG (1993). Laser cavity for generation of variable-radius rings of light. Opt. Lett..

[5] Lee HS (1994). Holographic nondiverging hollow beam. Phys. Rev. A.

[6] Yin J (1997). Generation of a dark hollow beam by a small hollow fiber. Opt. Commun..

[7] Paterson C, Smith R (1996). Higher-order Bessel waves produced by axicon-type computer-generated holograms. Opt. Commun..

[8] Marksteiner S (1994). Coherent atomic waveguides from hollow optical fibers: quantized atomic motion. Phys. Rev. A.

[9] Balykin VI, Letokhov VS (1987). The possibility of deep laser focusing of an atomic beam into the A-region. Opt. Commun..

[10] Arlt J, Dholakia K (2000). Generation of high-order Bessel beams by use of an axicon. Opt. Commun..

[11] Cai Y, Lu X, Lin Q (2003). Hollow Gaussian beams and their propagation properties. Opt. Lett..

[12] Sun Q (2012). Hollow sinh-Gaussian beams and their paraxial properties. Opt. Express.

[13] Lin J (2013). Longitudinal polarized focusing of radially polarized sinh-Gaussian beam. Opt. Express.

[14] Liu Y, Kuang C, Liu X (2015). The use of azimuthally polarized sinh-Gauss beam in STED microscopy. J. Opt..

[15] Senthilkumar M (2019). Focusing properties of spirally polarized sinh Gaussian beam. Opt. laser Technol..

[16] Zou D (2017). Propagation properties of hollow sinh-Gaussian beams in quadratic-index medium. Opt. Commun..

[17] Ashkin A (1970). Acceleration and trapping of particles by radiation pressure. Phys. Rev. Lett..

[18] Ashkin A, Dziedzic JM, Bjorkholm JE, Chu S (1986). Observation of a single-beam gradient force optical trap for dielectric particles. Opt. Lett..

[19] Ashkin A (2000). History of optical trapping and manipulation of small-neutral particle, atoms, and molecules. IEEE J. Sel. Top. Quantum Electron.

[20] Harada Y, Asakura T (1996). Radiation forces on a dielectric sphere in the Rayleigh scattering regime. Opt. Commun..

[21] Ng J, Lin Z, Chan CT (2010). Theory of optical trapping by an optical vortex beam. Phys. Rev. Lett..

[22] Maragò OM (2013). Optical trapping and manipulation of nanostructures. Nat. Nanotechnol..

[23] Jiang Y (2016). Trapping two types of particles by modified circular Airy beams. Opt. Express.

[24] Tang B (2017). Radiation forces of beams generated by Gaussian mirror resonator on a Rayleigh dielectric sphere. 2017 Sci. Rep..

[25] Du J (2017). Tailoring optical gradient force and optical scattering and absorption force. Sci. Rep..

[26] Wang N (2018). Gradient and scattering forces of anti-reflection-coated spheres in an aplanatic beam. Sci. Rep..

[27] Chen M (2018). Optical force and torque on a dielectric Rayleigh particle by a circular Airy vortex beam. J. Quan. Spectrosc. Radiat. Transfer.

[28] Lu W (2019). Abruptly autofocusing property and optical manipulation of circular Airy beams. Phys. Rev. A.

[29] Collins SA (1970). Lens-System Diffraction Integral Written in Terms of Matrix Optics. J. Opt. Soc. Am..

[30] Erdelyi, A., Magnus, W. & Oberhettinger, F. *Tables of Integral Transforms*. (McGraw-Hill, NewYork. 1954).

[31] Wang, S. & Zhao, D. *Matrix Optics*. (CHEP-Springer, Beijing. 2000).

[32] Visscher K, Brakenhoff GJ (1992). Theoretical-study of optically induced forces on sphererical-particles in a single beam trap 1. Rayleight scatters. Optik.

